# The Role of Cell Membrane Information Reception, Processing, and Communication in the Structure and Function of Multicellular Tissue

**DOI:** 10.3390/ijms20153609

**Published:** 2019-07-24

**Authors:** Robert A. Gatenby

**Affiliations:** Departments of Radiology and Integrated Mathematical Oncology, Moffitt Cancer Center, Tampa, FL 33612, USA; robert.gatenby@moffitt.org

**Keywords:** information, signal conduction, cell membrane, genome, distributed system

## Abstract

Investigations of information dynamics in eukaryotic cells focus almost exclusively on heritable information in the genome. Gene networks are modeled as “central processors” that receive, analyze, and respond to intracellular and extracellular signals with the nucleus described as a cell’s control center. Here, we present a model in which cellular information is a distributed system that includes non-genomic information processing in the cell membrane that may quantitatively exceed that of the genome. Within this model, the nucleus largely acts a source of macromolecules and processes information needed to synchronize their production with temporal variations in demand. However, the nucleus cannot produce microsecond responses to acute, life-threatening perturbations and cannot spatially resolve incoming signals or direct macromolecules to the cellular regions where they are needed. In contrast, the cell membrane, as the interface with its environment, can rapidly detect, process, and respond to external threats and opportunities through the large amounts of potential information encoded within the transmembrane ion gradient. Our model proposes environmental information is detected by specialized protein gates within ion-specific transmembrane channels. When the gate receives a specific environmental signal, the ion channel opens and the received information is communicated into the cell via flow of a specific ion species (i.e., K^+^, Na^+^, Cl^−^, Ca^2+^, Mg^2+^) along electrochemical gradients. The fluctuation of an ion concentration within the cytoplasm adjacent to the membrane channel can elicit an immediate, local response by altering the location and function of peripheral membrane proteins. Signals that affect a larger surface area of the cell membrane and/or persist over a prolonged time period will produce similarly cytoplasmic changes on larger spatial and time scales. We propose that as the amplitude, spatial extent, and duration of changes in cytoplasmic ion concentrations increase, the information can be communicated to the nucleus and other intracellular structure through ion flows along elements of the cytoskeleton to the centrosome (via microtubules) or proteins in the nuclear membrane (via microfilaments). These dynamics add spatial and temporal context to the more well-recognized information communication from the cell membrane to the nucleus following ligand binding to membrane receptors. Here, the signal is transmitted and amplified through transduction by the canonical molecular (e.g., Mitogen Activated Protein Kinases (MAPK) pathways. Cytoplasmic diffusion allows this information to be broadly distributed to intracellular organelles but at the cost of loss of spatial and temporal information also contained in ligand binding.

## 1. Introduction

Living cells use information [1] and energy to maintain a stable ordered state far from equilibrium, allowing them to self-replicate and form functioning multi-cellular societies [2,3]. Since the information-encoding structure of DNA was demonstrated, virtually all investigation of information dynamics within the cells has focused on the genome. Gene networks are typically viewed as the primary regulators of cell function [4] similar to computing networks [5] so that the genome is typically modeled as a cellular central processor. Thus, the nucleus is usually viewed as the cellular “command center” [3] that receives, processes, and responds to all intra- and extra-cellular information.

This conventional model is clearly supported by the essential role of the nucleus in cell function and proliferation and the unambiguous mechanism for information storage in the triplet code of the DNA. This fundamentally motivates the intense and ongoing empirical investigation of the genome and gene networks as the primary governing dynamics in single cells and multicellular organisms. However, the implicit assumption that all cellular information is contained in the genome has not been validated by empirical observations. For example, in 2001, as the Human Genome Project neared completion, a betting pool named Genesweep [6] was formed so that scientists could wager their best estimate of the number of genes in the human genome. More than 400 scientists participated, with an average estimate of 66,000 ranging from 310,000 to 26,000 [6]. None were correct—the human genome, based on multiple different data sources, is now thought to contain fewer than 19,000 genes [7].

This somewhat embarrassing over-estimation of our species genome size was based on the widely accepted assumption that, as the sole repository of cellular information, the genome acts as a central processor [8,9] that exclusively receives, analyzes, and responds to all intracellular and extracellular signals. This concept of the nucleus as the cellular “control center” [10] is implicit in the expectation that the number of genes necessary for each organism must be some increasing function of the size and complexity of that organism. In fact, it is now clear that the human genome has roughly the same number of genes as worms and fewer genes than many species, including the frog and rice [11]. Failure of the prevailing paradigm to accurately predict the observed variations in genome size has led to a number of amendments that generally focus on complexity of genetic interactions. In essence, the human genome can do more with less through evolution of increasingly sophisticated gene networks and perhaps a greater role for miRNA [11]. Note, however, that in these discussions, the fundamental assumption that the genome is the site of all information dynamics that govern individual cell function and the formation of cellular societies with precise spatial organization and distribution of function to allow complex anatomic structure and physiological interactions in multicellular organisms.

An alternative hypothesis views the genome as simply one component of a broad multiscale, multimodal information dynamic within cells [12,13,14]. Specifically, the genome, while the sole source of heritable information, also produces the molecular machinery and physiological interactions that permit independent information storage, acquisition, and processing at other sites (the cell membrane, for example [15,16]) within the cell. This non-heritable information plays an essential role in the cell’s ability to interact with spatial and temporal variations in its environment. For single cells, this information includes global events, such as local changes in temperature as well as small perturbation, that just affect a single region of the cell surface. Similarly, some perturbations may occur and require adaptations over hours, days, or years while others occur in microseconds and require an equally fast response. In multicellular organisms, individual cells must respond to environmental signals that are, in effect, instructions regarding the location and differentiated state necessary for tissue structure and function. Thus, we might hypothesize the density and diversity of these non-genomic interactions between the cell membrane and the adjacent tissue environment may be a critical component in the development of size and complexity within multicellular organisms.

This hypothesis suggests that cellular information dynamics is a distributive system that at least involves the cell membrane and nucleus and most likely includes other organelles, such as the mitochondria, centrosome, and endoplasmic reticulum. This model is motivated by observations that the “nucleus as cellular command center” paradigm is subject to a number of practical and theoretical objections. Among them is the simple factor of time. A mammalian cell that is 20 microns in diameter is about 20,000 protein diameters across. Signals traveling from the cell membrane to the nucleus via pathway proteins diffusing in the three dimensions inevitably results in information degradation, since the location at which each protein arrives on the nuclear membrane and the time required for the transit are subject to considerable variations. Furthermore, cell responses to a critical perturbation often must occur in seconds or even microseconds—far too fast to allow communication to the nucleus, processing of information, synthesis of proteins, and transport of proteins to the critical site. Furthermore, feedback over time regarding the success or failure of the cell’s response requires continuous bidirectional communications, which will suffer the same degradation of information and time limitations noted above. In other words, if the nucleus is the sole location in which the cell can receive, process, and respond to information, the time required to send and receive signals from an acute, external perturbation would be excessively long. In contrast, we hypothesize that evolutionary optimization would select a distributed information system that includes a large repertoire of information receptors and response modules that allow rapid and spatially specific responses to the diverse external and internal signals and perturbations a cell will receive in its lifetime.

## 2. Is the Information Stored in the Genome Sufficient for Cellular Order and Function?

An apparent gap between the information content of a cell and that of the genome and its products has been previously noted [13,17,18]. Over six decades ago, Morowitz [17,18] calculated the complexity of the three-dimensional structures in a single *Escherichia Coli* requires about 2 × 10^11^ bits of information. A similar estimate is obtained using a calorimetric approach. In contrast, the information storage capacity of the *E. Coli* genome is 10^7^ bits [17]. Of course, the genomic information is expanded through many translations so that one gene can be amplified to hundreds or thousands of proteins. The total information content of genes and gene products can be estimated because the average molecular composition of *E coli* is known. The mRNA content is four-fold larger than the DNA, and the typical protein content of *E. coli* is 1.6 × 10^−13^ g/cell. Assuming the average weight of amino acids is 110, about 8.4 × 10^8^ amino acids are incorporated into the intracellular proteins of *E coli*. Using Shannon information, 4.2 bits of information are gained with each amino acid for a total information content of protein of about 3.4 × 10^9^ bits. Thus, the total information content contributed to the *E Coli* from the genome is about two orders of magnitude smaller than the information content of the cell. In other words, most of the information in a cell is not a gene or gene product [13].

Actually, this exercise simply restates the obvious. There are clearly ordered structures in the cell other than proteins and polynucleotides. Membranes, for example, constitute about 60% of the cell mass and contain about 10^9^ lipid and glycolipid molecules. In mammalian cells, over 200 different species of lipid molecules contribute to the membrane, and their relative content is precisely controlled—varying among different cell types, different regions of the same cell and even in the inner and outer layers of the nuclear membrane [13]. The information content of membranes in a “typical” mammalian cell has been calculated to be on the order of 5 × 10^10^ bits [13,17]. While proteins catalyze the formation of lipids, this feed-forward dynamic is inherently unstable, and clearly other mechanisms must play a critical role in controlling the lipid distribution.

A somewhat less obvious information-containing structure is transmembrane ion gradients [15]. The asymmetric distribution of Na^+^, Cl^−^, Ca^+^, and K^+^ across cell membranes (manifesting as transmembrane potentials in the range of −80 to −40 mV [19]) as well as the H^+^ gradient across the mitochondria represent highly ordered (i.e., non-random) structures. The critical role of the transmembrane gradient in the biology of eukaryotic cells is evidenced by observations that up to 40% of the cell’s energy budget is used to maintain them [20].

## 3. What is Information in the Context of Living Systems?

In one of the most famous *gedankens* (thought experiments) in scientific history [21], Maxwell proposed a remarkably clever set of initial conditions [22]: Two closed boxes at identical temperatures containing the same gas are connected by a channel in which a “demon” operated a frictionless gate. Because the gas molecules have a distribution of velocities, the demon could allow only faster molecules to move from one box to another and slower molecules in the opposite direction. This would generate an apparently spontaneous heat flow between regions of the same temperature—a violation of the first law of thermodynamics.

In the early 20th Century, Szilard [23] and others resolved this conundrum by pointing out the demon forced the heat flow by using information (i.e., the velocity of the molecules) [24]. This was the birth of information theory, which reached maturity in Shannon’s seminal work [25] on communication that first quantified information in “bits”.

In 1970, Johnson, writing in Science [26], predicted information theory would become the “calculus of biology”. Although it is clear that information is necessary for living systems, information theory thus far has contributed little to biology and certainly has not become its primary theoretical framework. In fact, even the definition of information in a biological context remains controversial. Since the mathematics of information theory rely on probability distributions, information can be viewed as a deviation from randomness or the degree of “unexpectedness” [13,14]. More importantly, the connection of information to order and stability in living systems remains unclear. In some ways the challenge is evident in the very metric of information contents—bits. The elegant mathematical basis for this metric has been clear for decades, and the predicted link between information [25] and thermodynamic energy has been empirically confirmed [27]. However, bits may provide a metric for quantity of information, they say nothing about meaning (i.e., context) and provide no clear mechanism by which information is converted to thermodynamic work, reduces cellular entropy, and increases cellular order [2].

**Figure 1:**
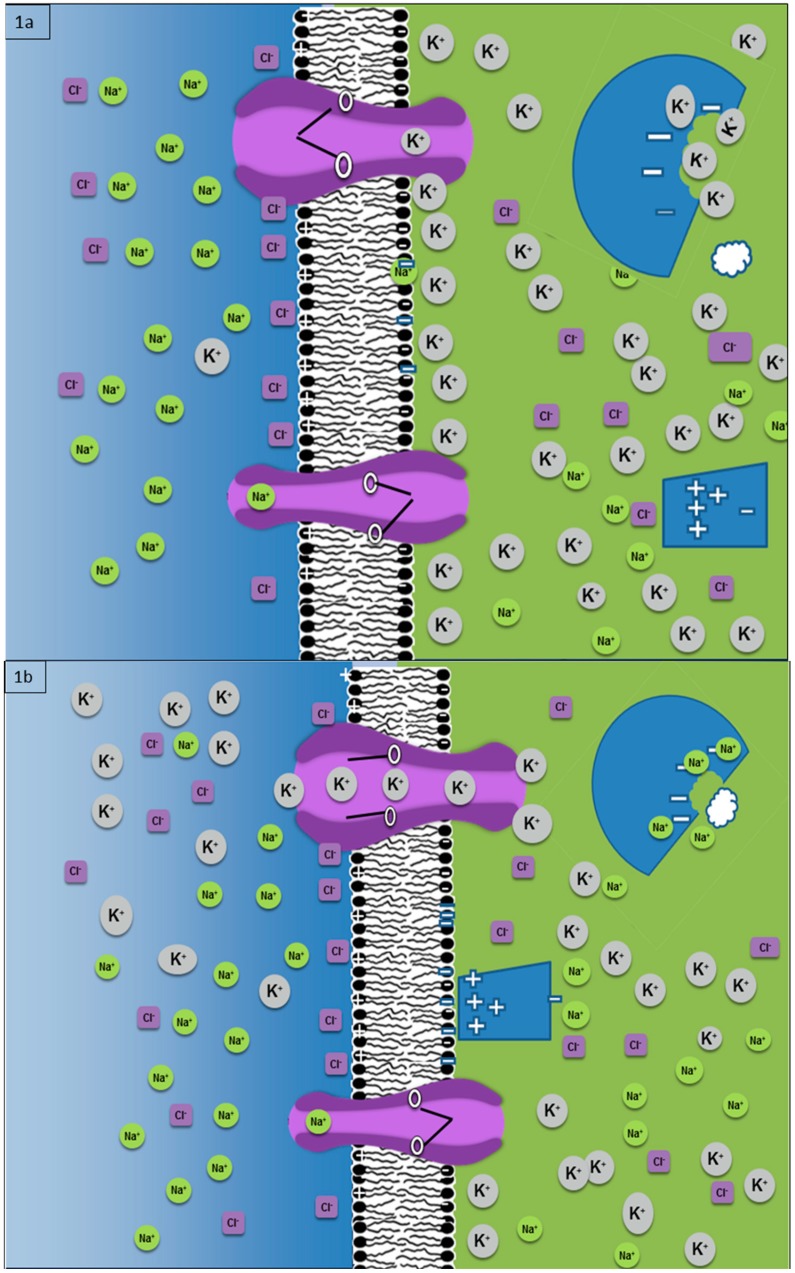
Information detected by the protein gate in a K+ channel in the cell membrane opens the gate allowing rapid efflux of most abundant mobile cation. (**a**) Baseline state with large transmembrane gradients in Na^+^, Cl^−^, and K^+^ generated by ATP-dependent membrane pumps. In the cytoplasm (K^+^), as the mobile cation with the highest concentration, is the primary shield of negative charges on the inner leaf of the cell membrane and reaction pocket of an enzyme (this neglects Ca^2+^ and Mg^2+^, which can also serve as cation shields but are present in very low concentrations). The active site of the enzyme is too large to match the shape of the substrate, and the shielding of the inner leaf of the membrane does not allow it to attract the positive charges on the peripheral membrane protein. (**b**) Following an external perturbation that opens the K^+^ channel, a rapid efflux of K^+^ briefly lowers the K^+^ concentration in the adjacent cytoplasm. This reduces the shield on the negative inner leaf of the cell membrane allowing positively charged peripheral membrane protein. This reduction in shielding of the inner leaf of the membrane may also cause local changes in the lipid asymmetry in the cell membrane (e.g., negatively charged phosphatidylserine normally in the inner leaf) which can alter membrane stiffness or release of lipid second messengers, such as PIP3. The change in cation concentration alters the reaction site on the enzyme so that it can now bind the adjacent substrate and catalyze the reaction. This dependence of protein function on cation concentrations has been observed empirically. We speculate a cause may be related to the smaller volume Na^+^ ions compared to K^+^ ions.

In living systems, information is useful only when it is converted to order or thermodynamic work. Gatenby and Frieden [28] proposed one mechanism by which living systems convert unit-less bits stored in the genome to thermodynamic work, and “meaning” (or specificity) is apparent in proteins that serve as enzymes. The amino acid sequence that forms the protein is encoded unambiguously in the genetic DNA, and this information is converted to a three-dimensional structure as the protein folds into its lowest free energy state. Thus, the 3D configuration is specified by the gene information, since the folding is dependent on the interaction of the amino acids in the protein. We propose this folding process represents an increase in order and information, which is expressed thermodynamically as a release of free energy as the protein assumes a minimum free energy state. Importantly, this conversion process is subject to modification by environmental information in the form of temperature, pH, ion concentrations, and so on. Of particular interest are the variations in concentrations of mobile cations on protein functions. Page and Di Cera [29] demonstrated empirically the activity of many important intracellular enzymes is dependent on the local concentration of Na^+^ and K^+^. The role of Ca^2+^ concentrations for kinase activity [30] has been extensively investigated. Other enzymes, hexokinase and phosphofructokinase, for example, are dependent on Mg^2+^ concentrations [31]. These observations are the outcomes of interactions of cations with negative charges on some amino acids in the protein. But it is likely similar dynamics will produce many other biologically important changes in macromolecules. Furthermore, as demonstrated in Figure 1, cations shield negative charges on the inner leaf of the cell membrane while mobile anions can shield positive charges on the nuclear membrane and intracellular structures. Brief ion fluxes have the effect of uncovering these charges allowing coulomb interactions with adjacent macromolecules.

In enzymes, the acceleration of a reaction requires the shape of the catalytic pocket to match that of the substrate (like a “key and lock”). As noted above, the enzyme shape is largely dependent on the genetic amino acid sequence but also subject to fluxes in local environmental factors. The degree of matching of the catalytic pocket to the substrate can be quantified by the Kullback–Leibler (K–L) divergence [32,33,34], which is also a metric of information. Because this spatial match and corresponding K–L Divergence for each enzyme is unique to each substrate, these dynamics add specificity to the information content of the gene. In other words, the information content of each enzyme in bits, as described by the K–L Divergence, is not fixed but rather dependent on the substrate with which it is attempting to bind. If the substrate key does not fit into the enzyme lock, there is no informational effect in that transaction. Finally, we note these dynamics allow observation and quantitation of the conversion of information to thermodynamic work through the reduction of the activation energy. Note this does not alter the initial or final energy state of the reaction but, rather, alters the speed with which the reaction occurs. Remarkably, enzymes can increase the rate of reaction by as much as 15 orders of magnitude! To support this theoretical model, we note the Arrhenius equation, which was developed solely through empirical observations, can be theoretically derived using K–L Divergence [28].

## 4. Information Dynamics in the Cell Membrane

As noted above, the membrane structure and associated transmembrane ion gradients represent a large storehouse of information. How is that used by the cell to maintain order? The answer is observable in a common experience when walking toward the entrance of a heated building on a cold day. Should the door open as you approach it from inside the building, you will feel a rush of warm air. Note that, even if you are not looking at the door, the release of warm air along the thermal gradient between the building and environment is clear evidence that it has opened. If the door was linked to a sensor so that it opened upon some perturbation, the flow of warm air is transmission of information arriving at the sensor into the building where it can be received and processed (by you and others) as a puff of warm air similar to ion puffs observed in cells [35]. Note that, assuming the door immediately closes, this flow of information into the building in the form of warm air will be localized and brief as the temperature gradient rapidly dissipates with distance from the door and with time following the opening.

Cells generate a similar transmembrane ion gradient to the thermal gradient described above. Here we will focus on the analogous series of events when the gate on a transmembrane ion channel opens in response to a perturbation allowing rapid flow of ions along the pre-existing transmembrane concentration gradients constantly maintained by eukaryotes. This process is well known in the propagation of depolarization waves [36] along a neuron (described by the well-known Hodgkin–Huxley equation [37]). This led to the hypothesis that these dynamics represent a specialized application of membrane information processing and transmission in the plasma membranes of non-neuronal cells [15]. Several hundred different types of gates (e.g., voltage-dependent) for membrane ion channels are encoded in the human genome. Each can act as signal receptors. When the gate opens, ions flow rapidly along the membrane channels. This represents transmission of information received by the gate into the cell. This has been visualized experimentally as Ca^2+^ ion puffs [35]. Although these puffs disperse quickly, they will briefly alter the local ion concentrations in the cytoplasm adjacent to the channel. This information is then “processed” (Figure 1) as the change in cations alters enzyme function and the loss of shielding of negative charges on the inner leaf of the membrane can both attract positively charged macromolecules and (possibly) the local distribution of phospholipids in the cell membrane. Furthermore, there is the possibility of propagating a depolarization wave along some or all of the adjacent membrane [35].

Mathematical models of ion flow through transmembrane channels has demonstrated this to be a highly efficient process that minimizes the loss of signal [15]. There are several hundred different protein gates that can detect and respond to a wide range of environmental signals. When the gate opens, the large, pre-existing transmembrane ion gradients allow immediate and rapid flow (about 10^7^ per second [38]) of a specific anion or cation (each channel is highly ion-specific) into or out of the cell. These dynamics are identical to those associated with propagation of a depolarizing wave along neurons as described by the Hodgkin–Huxley equation [37]. Recently, we have, in fact, demonstrated the Hodgkin–Huxley equation can be derived from first principles of information theory used to model the flow of information across the membrane of non-neuron cells [15]. This supports the hypothesis that the transmembrane ion flows that produce a travelling depolarization wave in neurons are simply a specialized adaptation of membrane information dynamics found universally in eukaryotic cells.

How do cells process and respond to information transmitted by transmembrane ion flows? The key to this is likely the above-noted sensitivity of proteins to local ion concentrations. By altering the function and location of peripheral membrane proteins at the site of the ion flux, the cell can rapidly and locally respond to many perturbations (Figure 1). It is likely that similar dynamics will occur in the extracellular space adjacent to the channel. Furthermore, since changes in the ion concentrations dissipate rapidly over space and time, these dynamics allow the cell to repeatedly assess critical spatial and temporal information. Thus, environmental signals that are time-dependent and/or spatially localized can be resolved within the membrane. For single-cell eukaryotes, this allows rapid response to critical environments, such as the presence of a dangerous predator or a potential meal. For eukaryotic cells that are part of a multicellular organism, these dynamics can promote a response to sudden perturbation, such as an injury, but are also likely required to use spatial and temporal signals from the local tissues that convey instructions on the required cellular location and function within that tissue.

## 5. Information Transmission within Cells

**Figure 2:**
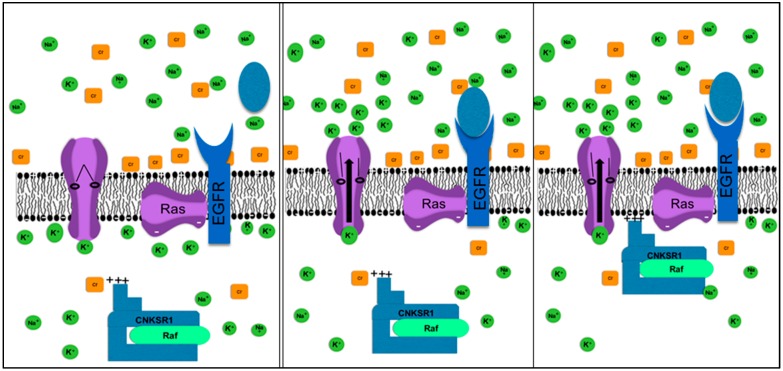
The role of ion dynamics in EGFR signaling. Ligand binding to EGFR activates Ras through binding of GTP (adding negative charges). Empirical data then show rapid movement, then show “Ras recruits Raf to the membrane” [43] leading to signal transduction to the nucleus along the MAPK pathways. The latter, however, provides no information of the physical mechanism. A hypothesis is that the K^+^ channel associated with EGFR opens upon ligand binding. The outflow of K^+^ reduces shield on the inner leaf of the membrane. Raf, with a pK of 9.3, is positively charged, and there are clustered positive charges on the scaffolding protein CNKSR1. These allow coulomb interactions with negative charges on the inner leaf of the cell membrane resulting in the physical movement observed experimentally.

**Figure 3:**
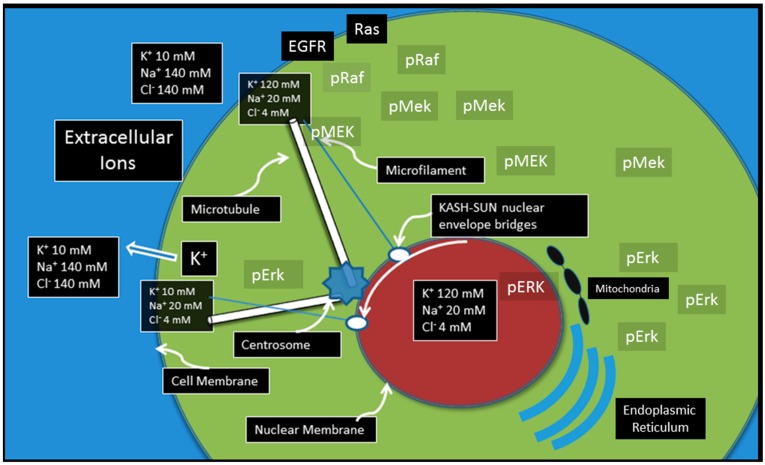
An integrative model of communication from cell membrane receptors to other components of the cell. The canonical molecular pathways are amplifiers that allow signals to reach the nucleus, mitochondria, and endoplasmic reticulum. However, diffusion in three dimensions results in degradation of spatial and temporal information. The microfilaments and microtubules allow rapid, bi-directional flow ions between the cytoplasm adjacent to the cell membrane and the centrosome (via microtubules) and the nuclear membrane (via microfilaments). This permits rapid communication of both coarse-grained and fine-grained information with minimum information loss. Furthermore, the associated spatial and temporal information may be critical for survival in single-cell organisms and in the organization and function of multicellular tissue.

The cell membrane, as the interface between a cell and its environment, is the site at which most environmental information is received. As noted above, some perturbations require only a local response. However, some require an integrated reaction from multiple cellular components. Thus, communication from the cell membrane to the nucleus and other components of the cell is critical. This is summarized in Figure 2 and Figure 3.

### 5.1. The Canonical Molecular Pathways

One mechanism of intracellular information transmission is that of the molecular pathways. Following binding of a ligand to a membrane receptor, pathway proteins (e.g., MAPK pathway [39]) are typically phosphorylated and move through the cytoplasm to convey the information to the nucleus resulting in changes in gene expression. Importantly, however, these pathway proteins also interact with other intracellular organelles, such as the mitochondria, endoplasmic reticulum, cytoskeleton, and centrosome, all of which are encapsulated by their own membranes with inherently different transmembrane ion gradients than the cell and nuclear membranes. Accordingly, these differing transmembrane informational gradients further support dynamic information processing at various distributed locations within the cell using the same fundamental mechanisms as found in the cell membrane.

These information pathways are widely investigated, and dysfunction in membrane receptors and pathway proteins is present in the vast majority of human cancers. In general, these pathways act as amplifiers as each kinase in the pathway expands the signal by producing multiple phosphorylated proteins. Importantly, however, the primary model of transit, diffusion, in three dimensions is notoriously wasteful of time and energy, as evidenced by the joking comment attributed [40] to Yale mathematician, Shizuo Kakutanni, that “a drunk man will find his way home, but a drunk bird may get lost forever”. On a practical level, the variability of signal amplification by the inevitably imprecise kinase activation as well as the limitation of 3D diffusion significantly degrade the information content of the pathway signals [41,42].

A critical component of this information pathway is the transmission of signal from membrane bound Ras (Figure 2) to Raf, which can then diffuse through the cytoplasm. Experimentally, addition of GTP to Raf following ligand binding to an Epidermal Growth Factor Receptor (EGFR) occurs much more quickly than expected by simple diffusion. This is often described as “Ras recruits Raf to the membrane” [43], although this common phrase actually provides no information on the physical mechanism of this movement. As shown in Figure 2, EGFR activates K^+^ channels [44] and the resulting outflow of cations decrease shielding on the inner leaf of the cell membrane and the Raf-GTP complex allowing movement of positively charged Raf and its associated scaffolding protein.

Thus, it appears that the role of molecular pathways signal transmission is to carry information broadly to all components of the cell. Since lossy (due to degradation of spatial and temporal information noted above) information is sufficient, we can assume that the pathways carry signals that are critical but not time dependent. In particular, the pathways carry signals that influence the global state of the cell. That is, in unicellular organisms, ligand binding appears to be informing each cell about the relative abundance of similar species in its locality to facilitate mating and loose group dynamics in the event of insufficient local resources. In cells within a multicellular organism, the pathways carry instructions regarding the general state of the cell (e.g., proliferate, die, remain quiescent).

### 5.2. Transmitting Spatial and Temporal Information from the Cell Membrane

Many studies have demonstrated that the main elements of the cytoskeleton (microtubules and microfilaments), in addition to their structural role in cell shape and movement, have the capacity to conduct ions [45,46,47,48,49]. This seems likely to be a potential mechanism of signal transmission [47,49], but the biological role for this conduction has not been clearly demonstrated. We propose that, while much the information received at the membrane can be processed locally to produce only a local response, some signals may require a global cellular response and, thus, must be disseminated to the nucleus and other organelles.

This led to the hypothesis that communication to and from the cell membrane can be carried by elements of intracellular cytoskeleton [16]. Both microtubules and microfilaments conduct ions and are highly dynamic structures that often extend their distal tip to the cytoplasm adjacent to the cell membrane. Proximally, the microtubules typically converge on the centrosome [47,50,51] while microfilaments attach to LINC proteins on the nuclear membrane [52,53]. The cytoskeleton also provides links to the mitochondria [54] and endoplasmic reticulum [55]. The biomechanical role of the cytoskeleton is well documented and, in particular, the microfilament connections to the LINC proteins [56] govern localization and movement of the nucleus and, through Laminin A, can alter both gene transcription and chromosomal location. However, since both elements of the cytoskeleton form wire-like structures that can conduct ionic signals, it seem reasonable to hypothesize that they can transmit information from cytoplasmic perturbation in ion concentrations to the nucleus and centrosome. Mathematical analysis of information conduction by the cytoskeleton has demonstrated that the larger microtubules (about 25 nm in diameter) optimally carry coarse-grained information while the small microfilaments (about 3 nm in diameter) carry fine-grained information [16]. These modes of information conduction provide a greater dynamic range that permits the cell to optimally maintain a complete and accurate assessment of its surrounding environment [16] (Figure 3).

## 6. Conclusions

The conceptual model of the nucleus as a cell’s central processor and control center is anthropomorphic and, therefore, intuitively appealing, but the temporal and spatial demands of cellular functions suggest this would be a suboptimal organizing principle. To be clear, heritable information in the genome is necessary to generate all components of this information network. That is, gene-encoded proteins are membrane pumps and detectors that generate, similar to Maxwell’s Demon, a large transmembrane ion gradient as well as the peripheral membrane proteins that process and respond to local signals. However, once formed, the membrane information processor can function as a critical and, at times, independent component within a broad, integrated cellular information network. It is linked to the nucleus and other organelles by a highly sophisticated communication system embedded in the cytoskeleton allowing synchronous response to some perturbations. However, the membrane information processor can also process and respond to local perturbations independent of additional input from the nucleus.

Perhaps the best evidence for the critical role of the transmembrane ion gradient in a distributed information system is simply the energy cells used to maintain it. Guppy et al. [20] found that up to 40% of a mammalian cell’s energy budget is devoted to maintaining the transmembrane ion gradient. Since evolution is a relentless optimization process that continuously balances cost and benefit, one can generally assume that the energy devoted to a cellular function is a good estimate of its evolutionary value.

Additional support is found in considering the time requirement imposed by the assumption that the genome is the central processor and the nucleus the cellular control center. External information frequently requires actions on a timescale too short to allow communication to the membrane to the DNA, processing, decision making, and subsequent protein synthesis and delivery to the site of a perturbation. On the other hand, a distributed information system permits parallel “computing” and maximal efficiency. Furthermore, channels in the cell membrane allow eukaryotic cells to acquire spatial and temporal information from the environment with high resolution. This information can be communicated to other organelles through both fine-grained and coarse-grained conduits. This augmentation of information generated by cell membrane receptors and carried by diffusion through molecular pathways allows single-cell eukaryotes to respond quickly to threats or opportunities. In multicellular organisms, membrane information dynamics may be critical for proper distribution of cells in spatially complex structures as well as synchronized function of those cells necessary for optimal tissue function.

Finally, the hypothesized spatial and temporal information in the membrane will likely be critical in determining the robust spatial organization of cells necessary for functioning tissue within multicellular organisms. Indeed, if the spatial organization and function of multicellular tissue is primarily governed by information dynamics in the membrane, the lack of correlation between organism complexity and genomic size is much less surprising. If so, this hypothesis generates a straightforward prediction: The number and distribution of transmembrane ion channels and intracellular signaling networks will increase as the complexity of an organism increases. This prediction can be tested through empirical observations.

## References

[1] Farnsworth K.D., Nelson J., Gershenson C. (2013). Living is information processing: From molecules to global systems. Acta Biotheor..

[2] Gatenby R.A., Frieden B.R. (2013). The critical roles of information and nonequilibrium thermodynamics in evolution of living systems. Bull. Math. Biol..

[3] Gatenby R.A., Frieden B.R. (2007). Information theory in living systems, methods, applications, and challenges. Bull. Math. Biol..

[4] Bota M., Dong H.W., Swanson L.W. (2003). From gene networks to brain networks. Nat. Neurosci..

[5] Yan K.K., Fang G., Bhardwaj N., Alexander R.P., Gerstein M. (2010). Comparing genomes to computer operating systems in terms of the topology and evolution of their regulatory control networks. Proc. Natl. Acad. Sci. USA.

[6] Roberts L. (1991). Gambling on a shortcut to genome sequencing. Science.

[7] Zhang J. (2007). The drifting human genome. Proc. Natl. Acad. Sci. USA.

[8] Shapiro J.A. (2006). Genome informatics: The role of DNA in cellular computations. Biol. Theory.

[9] D’Onofrio D.J., An G. (2010). A comparative approach for the investigation of biological information processing: An examination of the structure and function of computer hard drives and DNA. Theor. Biol. Med. Model..

[10] Cooper G.M., Hausman R.E. (2013). The Cell: A Molecular Approach.

[11] Pray L. (2008). Eukaryotic genome complexity. Nat. Educ..

[12] Frieden B.R., Gatenby R.A. (2011). Information dynamics in living systems: Prokaryotes, eukaryotes, and cancer. PLoS ONE.

[13] Gatenby R.A., Frieden B.R. (2005). The role of non-genomic information in maintaining thermodynamic stability in living systems. Math. Biosci. Eng..

[14] Stern R.G., Milestone B.N., Gatenby R.A. (1999). Carcinogenesis and the plasma membrane. Med. Hypotheses.

[15] Gatenby R.A., Frieden B.R. (2017). Cellular information dynamics through transmembrane flow of ions. Sci. Rep..

[16] Frieden B.R., Gatenby R.A. (2019). Signal transmission through elements of the cytoskeleton form an optimized information network in eukaryotic cells. Sci. Rep..

[17] Morowitz H.J. (1955). Some order-disorder considerations in living systmes. Bull. Math. Biophys..

[18] Morowitz H.J. (1968). Energy Flow in Biology: Biological Organization as a Problem in Thermal Physics.

[19] Albert B., Johnson A.D., Lewis J., Morgan D., Raff M., Roberts K., Walter P. (2014). Molecular Biology of the Cell.

[20] Guppy M., Kong S.E., Niu X., Busfield S., Klinken S.P. (1997). Method for measuring a comprehensive energy budget in a proliferating cell system over multiple cell cycles. J. Cell. Physiol..

[21] Shenker O.R. (2004). Maxwell’s demon 2: Entropy, classical and quantum information, computing. Stud. Hist. Philos. M. P..

[22] Weinstein S. (2003). Objectivity, information, and maxwell’s demon. Philos. Sci..

[23] Devereux M. (2003). A modified szilard engine: Measurement, information, and maxwell’s demon. Found. Phys. Lett..

[24] Rex A. (2017). Maxwell’s demon-a historical review. Entropy.

[25] Shannon C.E. (1959). A mathematical theory of communication. Bell System Tech. J..

[26] Johnson H.A. (1970). Information theory in biology after 18 years. Science.

[27] Bérut A., Arakelyan A., Petrosyan A., Ciliberto S., Dillenschneider R., Lutz E. (2012). Experimental verification of landauer’s principle linking information and thermodynamics. Nature.

[28] Gatenby R., Frieden B.R. (2016). Investigating information dynamics in living systems through the structure and function of enzymes. PLoS ONE.

[29] Page M.J., Di Cera E. (2006). Role of Na+ and K+ in enzyme function. Physiol. Rev..

[30] Braun A.P., Schulman H. (1995). The multifunctional calcium/calmodulin-dependent protein kinase: From form to function. Annu. Rev. Physiol..

[31] Garfinkel L., Garfinkel D. (1985). Magnesium regulation of the glycolytic pathway and the enzymes involved. Magnesium.

[32] Kullback S., Cornfield J. (1976). An information theoretic contingency table analysis of the dorn study of smoking and mortality. Comput. Biomed. Res..

[33] Kullback S., Leibler R.A. (1951). On information and sufficiency. Ann. Math. Stat..

[34] Ireland C.T., Kullback S. (1968). Minimum discrimination information estimation. Biometrics.

[35] Swillens S., Dupont G., Combettes L., Champeil P. (1999). From calcium blips to calcium puffs: Theoretical analysis of the requirements for interchannel communication. Proc. Natl. Acad. Sci. USA.

[36] Hodgkin A.L., Huxley A.F., Katz B. (1952). Measurement of current-voltage relations in the membrane of the giant axon of loligo. J. Physiol..

[37] Hodgkin A.L., Huxley A.F. (1952). A quantitative description of membrane current and its application to conduction and excitation in nerve. J. Physiol..

[38] Milo R., Phillips R. (2015). How many ions pass through an ion channel per second?. Cell Biology by the Numbers.

[39] Zhang W., Liu H.T. (2002). Mapk signal pathways in the regulation of cell proliferation in mammalian cells. Cell Res..

[40] Durrett R. (2019). Probability: Theory and examples.

[41] Cunningham J., Estrella V., Lloyd M., Gillies R., Frieden B.R., Gatenby R. (2012). Intracellular electric field and ph optimize protein localization and movement. PLoS ONE.

[42] Gatenby R.A., Frieden B.R. (2010). Coulomb interactions between cytoplasmic electric fields and phosphorylated messenger proteins optimize information flow in cells. PLoS ONE.

[43] Marais R., Light Y., Paterson H.F., Marshall C.J. (1995). Ras recruits raf-1 to the plasma membrane for activation by tyrosine phosphorylation. EMBO J..

[44] Peppelenbosch M., Tertoolen L.G.J., de Laat S.W. (1991). Epidermal growth factor-activated calcium and potassium chan. J. Biol. Chem..

[45] Hunley C., Uribe D., Marucho M. (2018). A multi-scale approach to describe electrical impulses propagating along actin filaments in both intracellular and in vitro conditions. RSC Adv..

[46] Patolsky F., Weizmann Y., Willner I. (2004). Actin-based metallic nanowires as bio-nanotransporters. Nat. Mater..

[47] Priel A., Ramos A.J., Tuszynski J.A., Cantiello H.F. (2006). A biopolymer transistor: Electrical amplification by microtubules. Biophys. J..

[48] Priel A., Tuszynski J.A. (2008). A nonlinear cable-like model of amplified ionic wave propagation along microtubules. EPL (Europhys. Lett.).

[49] Woolf N.J., Priel A., Tuszynski J.A. (2009). The cytoskeleton as a nanoscale information processor: Electrical properties and an actin-microtubule network. Nanoneuroscience. Biological and Medical Physics, Biomedical Engineering.

[50] Doxsey S. (2001). Re-evaluating centrosome function. Nat. Rev. Mol. Cell Biol..

[51] Petry S., Vale R.D. (2015). Microtubule nucleation at the centrosome and beyond. Nat. Cell Biol..

[52] Horn H.F. (2014). Linc complex proteins in development and disease. Curr. Top. Dev. Biol..

[53] Ostlund C., Folker E.S., Choi J.C., Gomes E.R., Gundersen G.G., Worman H.J. (2009). Dynamics and molecular interactions of linker of nucleoskeleton and cytoskeleton (linc) complex proteins. J. Cell Sci..

[54] Boldogh I.R., Pon L.A. (2006). Interactions of mitochondria with the actin cytoskeleton. Biochim. Biophys. Acta.

[55] Gurel P.S., Hatch A.L., Higgs H.N. (2014). Connecting the cytoskeleton to the endoplasmic reticulum and golgi. Curr. Biol..

[56] Starr D.A., Fischer J.A. (2005). Kash’n karry: The kash domain family of cargo-specific cytoskeletal adaptor proteins. Bioessays.

